# The Development and External Validation of Artificial Intelligence-Driven MRI-Based Models to Improve Prediction of Lesion-Specific Extraprostatic Extension in Patients with Prostate Cancer

**DOI:** 10.3390/cancers15225452

**Published:** 2023-11-17

**Authors:** Ingeborg van den Berg, Timo F. W. Soeterik, Erik J. R. J. van der Hoeven, Bart Claassen, Wyger M. Brink, Diederik J. H. Baas, J. P. Michiel Sedelaar, Lizette Heine, Jim Tol, Jochem R. N. van der Voort van Zyp, Cornelis A. T. van den Berg, Roderick C. N. van den Bergh, Jean-Paul A. van Basten, Harm H. E. van Melick

**Affiliations:** 1 Department of Urology, St. Antonius Hospital, 3435 CM Nieuwegein, The Netherlands; 2 Department of Radiation Oncology, Division of Imaging & Oncology, University Medical Center Utrecht, 3584 CX Utrecht, The Netherlands; 3 Magnetic Detection and Imaging Group, Technical Medical Centre, University of Twente, 7522 NH Enschede, The Netherlands; 4 Department of Radiology, St. Antonius Hospital, 3435 CM Nieuwegein, The Netherlands; 5 Department of Radiology, Canisius Wilhelmina Hospital, 7522 NH Nijmegen, The Netherlands; 6 Department of Urology, Canisius Wilhelmina Hospital, 7522 NH Nijmegen, The Netherlands; 7 Department of Urology, Radboud University Medical Center, 6525 GA Nijmegen, The Netherlands; 8 Quantib B.V., RadNet’s AI Division, 3012 KM Rotterdam, The Netherlands

**Keywords:** artificial intelligence, extraprostatic extension (EPE), machine learning, magnetic resonance imaging (MRI), prostate cancer (PCa), radiomics

## Abstract

**Simple Summary:**

The use of artificial intelligence algorithms can improve the prediction of lesion-specific histopathological extraprostatic extension (EPE) on MRI in prostate cancer patients. A lesion-specific prediction model can be helpful in counseling patients for radical prostatectomy and adequate preoperative information of the exact location of EPE may contribute to a total removal of the prostate cancer.

**Abstract:**

Adequate detection of the histopathological extraprostatic extension (EPE) of prostate cancer (PCa) remains a challenge using conventional radiomics on 3 Tesla multiparametric magnetic resonance imaging (3T mpMRI). This study focuses on the assessment of artificial intelligence (AI)-driven models with innovative MRI radiomics in predicting EPE of prostate cancer (PCa) at a lesion-specific level. With a dataset encompassing 994 lesions from 794 PCa patients who underwent robot-assisted radical prostatectomy (RARP) at two Dutch hospitals, the study establishes and validates three classification models. The models were validated on an internal validation cohort of 162 lesions and an external validation cohort of 189 lesions in terms of discrimination, calibration, net benefit, and comparison to radiology reporting. Notably, the achieved AUCs ranged from 0.86 to 0.91 at the lesion-specific level, demonstrating the superior accuracy of the random forest model over conventional radiological reporting. At the external test cohort, the random forest model was the best-calibrated model and demonstrated a significantly higher accuracy compared to radiological reporting (83% vs. 67%, *p* = 0.02). In conclusion, an AI-powered model that includes both existing and novel MRI radiomics improves the detection of lesion-specific EPE in prostate cancer.

## 1. Introduction

Adequate staging of prostate cancer (PCa) is essential for risk stratification and treatment assignment [1]. In local staging, it is important to accurately predict extraprostatic extension (EPE), which can be used to decide about the possibility of nerve-sparing prostatectomy. The neurovascular bundles lie adjacent to the prostate capsule, and preserving these nerves during radical prostatectomy is essential to reduce the risk of postoperative erectile dysfunction. However, nerve-sparing radical prostatectomy is independently related to an increased risk of positive surgical margins, which in turn poses a potential risk of residual cancer and disease recurrence [2]. Accurate preoperative imaging is crucial for planning oncologically safe, nerve-sparing surgery and providing patients with informed expectations regarding erectile function recovery following (non) nerve-sparing radical prostatectomy.

Currently, 3 Tesla multiparametric magnetic resonance imaging (3T mpMRI) is the preferred diagnostic modality for local staging of PCa [3]. A meta-analysis showed that mpMRI has a high specificity of 90% but a limited sensitivity of 57% to detect EPE [4]. Conventional MRI-based radiomics for EPE prediction include the tumor diameter, apparent diffusion coefficients (ADC) from diffusion-weighted imaging (DWI), and tumor contact length (TCL) with the prostate capsule. Several studies [5,6,7] found that the TCL correlates with EPE, where most studies measured the maximum TCL on axial T2-weighted (T2w) images. It might be more accurate and comprehensive, however, to assess geometric radiomics that characterize the relationship between a lesion and the prostate capsule in a three-dimensional manner. For instance, the tumor contact surface area (TCSA) has been identified as an independent predictor of pathological EPE [8], indicating the importance of incorporating geometric radiomics into predictive models.

Recent studies [9,10,11,12] have explored radiomics-based artificial intelligence (AI) algorithms on prostate MRI and these showed promising results for EPE prediction at the prostate and prostate lobe levels. While many existing predictive models have relied on radiomics generated from manual segmentations of the prostate and lesions, we employed a semi-automated approach to generate geometric- and intensity-based radiomics for our predictive model. The aim of this study was to establish and validate a comprehensive AI-driven approach with innovative MRI radiomics and clinical variables for lesion-specific EPE predictions. 

## 2. Materials and Methods

### 2.1. Study Cohorts

The study population comprised patients with localized PCa undergoing robot-assisted radical prostatectomy (RARP) from two Dutch hospitals: St. Antonius Hospital (SAZ) and Canisius Wilhelmina Hospital (CWZ). Patients from SAZ were included if they underwent 3T mpMRI scans within twelve months prior to RARP from September 2014 to July 2021. Patients from CWZ were included if they underwent 3T prostate mpMRI scans either at CWZ or Radboud University Medical Center (Radboudumc) within twelve months prior to RARP from June 2016 to December 2021. The internal cohort comprised MRI data from SAZ and CWZ, and the external cohort included MRI data from Radboudumc. 

Patients with complete data on initial serum PSA level, MRI-based local staging, and histopathologic outcomes including ISUP grade group after prostate biopsy and RARP, surgical margin status (including positive surgical margin [PSM]), presence and location of EPE, and final histopathological stage were collected. From a total of 943 patients, 794 were included in the current study after the following exclusions: (a) image artifacts (n = 19), (b) software faults (n = 13), (c) no evidence of primary tumor on MRI according to radiology reporting (rT0) (n = 47), (d) pathological stage T2 lesion with positive surgical margin at lesion side (n = 67), (e) incomplete pathological data (n = 3). 

### 2.2. Data Collection

MRI data were acquired using a Siemens 3T MRI scanner (Magnetom Skyra, Siemens Healthineers, Erlangen, Germany) and a Philips 3T MRI scanner (Ingenia, Philips Healthcare, Best, The Netherlands), using a torso array RF coil. We included T2w and DWI sequences with corresponding ADC maps derived using vendor-supplied routines. Further details on the sequence settings are presented in Supplementary Table S1. MRI data were anonymized and exported to the network storage systems of the institutes. 

Radiology reporting was performed according to the Prostate Imaging Reporting and Data System (PI-RADS) v2 [13] by experienced uro-radiologists (>1000 prostate MRI reports) and MRI-based local staging was dichotomized at lesion-specific level. Lesions classified as organ-confined (radiological stage T2) with uncertain EPE were considered as non-EPE, consistent with the original report. Pathology reporting after RARP was collected as ground truth and histopathological correlation between MRI visible lesions and surgical pathology was extracted from the pathology reports. If EPE was observed, the laterality (left, right, or bilateral) and location (apical, anterior, lateral, posterolateral, posterior, bladder neck, or seminal vesicle invasion) were reported. This study incorporated MRI-targeted biopsy grades and, in cases where targeted biopsies were not performed, it included systematic biopsy grades from the ipsilateral side of the suspected lesion.

### 2.3. Segmentations

**Figure 1:**
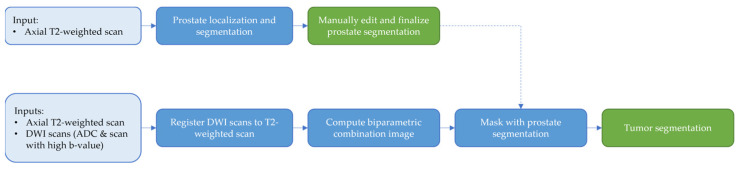
Flowchart demonstrating the tumor segmentation workflow. Input images, automatic processing steps, and manual steps are shown in light blue, blue, and green, respectively. Firstly, automatic prostate localization and segmentation is done using the T2-weighted scan. The user can perform any corrections deemed necessary and finalizes the segmentation. Secondly, the DWI scans are registered to the T2-weighted scan, after which the biparametric combination image is computed. The resulting biparametric combination image is masked with the finalized prostate segmentation. The masked biparametric combination image can then be used to identify and segment tumors.

Prostate and malignant lesions were segmented using the FDA-approved and CE-marked Quantib^®^ Prostate AI software (v1.3.0, Quantib B.V., Rotterdam, The Netherlands). Prostate segmentations were automatically computed by the AI software on axial T2w images and manually corrected and approved by the annotator (IB) under the supervision of two experienced uro-radiologists (EH, BC). Suspicious lesions in the peripheral and transition zone were semi-automatically identified on the biparametric combination image based on the T2w and DWI with corresponding ADC maps. The radiological reporting was reviewed to ensure the same suspicious lesions and their respective PI-RADS zone were identified. A flowchart of the segmentation workflow is shown in Figure 1. 

### 2.4. Radiomics

Radiomics, as a domain within medical imaging, aims to derive quantitative and multi-dimensional features from images through algorithmic approaches [14]. This process surpasses the limitations of conventional MRI features and enables the probing of complex tumor characteristics, such as the tumor diameter, tumor ADC characteristics, or tumor volume. 

**Figure 2:**
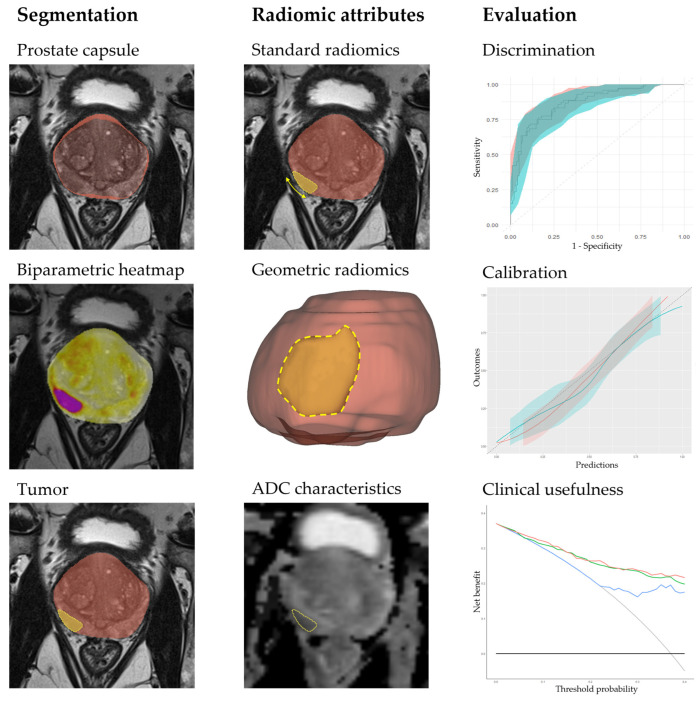
A schematic diagram illustrating the segmentations, the radiomic attributes, and the performance evaluation of the classification models. The segmentation process encompasses prostate segmentation on axial T2-weighted images and tumor segmentation on a biparametric combination image generated from T2-weighted and DWI data. The radiomic attributes include standard radiomics, such as tumor contact length (TCL), as well as novel geometric features such as tumor contact surface area (TCSA) and ADC characteristics.

In this study, geometric and MRI characteristics were extracted from the prostate and lesion segmentations using a custom image analysis toolkit (Python Software Foundation, Beaverton, OR, USA). We generated a 3D prostate capsule to calculate the length (TCL), surface area (TCSA), and volume (TCV) of contact between a lesion and the adjacent capsule. In addition, we calculated the contact surface area and volume using the Euclidean distances between the lesion surface and prostate capsule within 1 to 5 mm [8]. A schematic overview of the segmentation workflow, radiomic attributes, and model evaluation is shown in Figure 2. A comprehensive list of the MRI-based radiomics used in this study is provided in Supplementary Table S2.

### 2.5. Model Development

Three machine learning (ML)-classification models were developed, including random forest (RF), extra trees (ET), and logistic regression (LR), with histologically proven lesion-specific EPE as a binary outcome using the Scikit-learn Python library [15]. The lesions from the internal cohort were split into a training cohort (80%) and a test cohort (20%) after stratification by patient ID, to ensure that the patients with multiple lesions would be either in the training or the internal test cohort, and EPE to maintain the initial class balance. We used the Synthetic Minority Over-sampling Technique (SMOTE) [16] to oversample the EPE lesions in the training cohort to address data imbalance after stratification. In addition, the external cohort served as an independent validation set, designated as the external test cohort.

Clinical variables (i.e., PSA and ISUP biopsy grade) were added to the model and categorical variables (i.e., ISUP biopsy grade, lesion location) were encoded into numerical variables. Variables with correlations exceeding 95% were removed. Five-fold cross-validation was used for hyperparameter tuning and feature selection in the training cohort. Feature selection for RF and ET was done using impurity-based feature importance scores in Scikit-learn and feature selection for LR was determined with elastic net regression. 

### 2.6. Model Evaluation

The accuracy, sensitivity, specificity, positive predictive value (PPV), negative predictive value (NPV), and area under the curve (AUC) of the receiver operating characteristic (ROC) were evaluated on the internal and external test cohorts. The robustness of the models was assessed by varying the geometric variables with 5% higher and lower values. McNemar tests were performed to compare the accuracy, sensitivity, and specificity between our models and the radiologists, and the relative predictive value method [17] was applied to compare the PPVs and NPVs. Model calibration was performed to assess the agreement between observed endpoints and predictions using the runway package [18]. The clinical usefulness of the models was evaluated using the decision curve analysis (DCA) [19]. Statistical analyses were conducted using R (v4.1, R Foundation for Statistical Computing, Vienna, Austria), and significance was set at *p* ≤ 0.05.

## 3. Results

### 3.1. Participants

Overall, 994 lesions established using preoperative 3T mpMRI of 794 RARP patients were analyzed. The training, internal test, and external test cohorts consisted of 643 lesions (236 EPE and 407 non-EPE), 162 lesions (60 EPE and 102 non-EPE), and 189 lesions (71 EPE and 118 non-EPE), respectively. Baseline characteristics of the study population are shown in Table 1. 

### 3.2. Model Performance

#### 3.2.1. Discrimination

The performance measures of the classification models are presented in Table 2. The AUC of the models ranged from 0.86 to 0.91 at the lesion-specific level. In the external test cohort, the sensitivity was highest for ET with a value of 0.79 (95% CI 0.68–0.87), followed by RF with a sensitivity of 0.72 (95% CI 0.60–0.81), and LR with a sensitivity of 0.65 (95% CI 0.53–0.75). In terms of specificity, the highest value was achieved by LR with a specificity of 0.93 (95% CI 0.87–0.97), followed by RF with a specificity of 0.89 (95% CI 0.82–0.93), and ET with a specificity of 0.82 (95% CI 0.74–0.88). In relation to geometric variation, the RF model showed the most robust performance, maintaining a sensitivity range between 0.69 and 0.73, while the LR model showed a range of 0.58 to 0.70 (Supplementary Table S3). The included features of the RF model are shown in Supplementary Figure S1.

#### 3.2.2. Calibration

**Figure 3:**
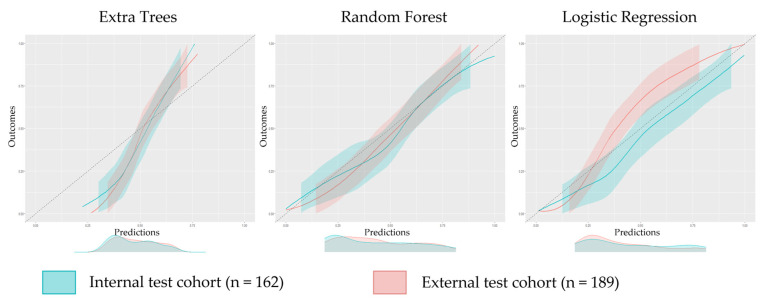
Calibration plots of the Extra Trees, Random Forest, and Logistic Regression model on the internal and external test cohorts at lesion-specific level.

The RF model demonstrated the highest agreement between predicted and observed probabilities of the internal and external test cohorts (Figure 3). The confidence intervals of the internal and external test cohorts showed substantial overlap, indicating comparable calibration performance in both datasets. The LR model demonstrated good calibration for the internal test cohort but showed underestimation beyond a risk threshold of 30%. The ET model exhibited poor calibration in both the internal and external test cohorts.

#### 3.2.3. Clinical Usefulness

**Figure 4:**
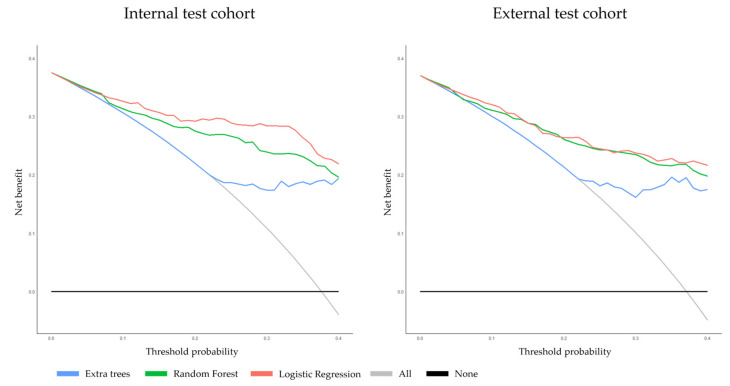
Decision curve analysis demonstrating the net benefit of the three prediction models on the internal and external test cohorts at lesion-specific level.

The net benefits of all models are presented within a clinically relevant range of risk thresholds of 0–40% (Figure 4). DCA revealed that the LR model yielded the highest net benefit, followed by the RF model, and the ET model. All models showed higher net benefits than “treat all” and “treat none” in risk thresholds between 0% and 40%. 

#### 3.2.4. Radiology Analysis

The radiological reporting performance measures are presented in Table 3. The RF model performed with statistically significantly better outcomes compared to the radiologists for sensitivity (72% vs. 53%, *p* = 0.02) and NPV (83% vs. 75%, *p* = 0.01) at the internal test cohort. At the external test cohort, the RF model showed significantly better performance in terms of specificity (89% vs. 69%, *p* < 0.001) and PPV (80% vs. 55%, *p* < 0.001). The accuracy was found to be statistically significantly higher for both the RF and LR models compared to the radiology reporting (83% vs. 67%, *p* = 0.02).

## 4. Discussion

This study showed that the use of AI-driven algorithms can improve the prediction of lesion-specific EPE on MRI in PCa compared to the present radiological reporting. By including a wide range of conventional and novel radiomics, an excellent discriminative performance at a lesion-specific level was found. The RF model showed good performance in calibration for both the internal and external test cohorts and was the most suitable option for individualized risk estimation among the three prediction models. It outperformed the radiology interpretations in terms of accuracy, specificity, and PPV in the external test cohort.

Our findings have important clinical implications. The superior discriminative ability suggests that this prediction model can potentially improve surgical planning. Improved preoperative detection of lesion-specific EPE can improve the safety of a nerve-sparing procedure and can potentially reduce the risk of positive surgical margins [20]. This can further positively impact the oncological outcomes of the surgical treatment of PCa, as positive surgical margins are known to be associated with disease recurrence [21]. 

Furthermore, our lesion-specific prediction model is the first step toward a lesion-specific indication for treatment assignment. Although progress has been made by adopting a side-specific approach of EPE prediction [22,23], instead of a whole-gland approach, predictions can be made more accurately when using a lesion-specific approach. The main advantage of the lesion-specific approach is that the geometric relation between the tumor and prostate capsule is considered. In this way, nerve-sparing indication can be evaluated more accurately, particularly in anteriorly located tumors where the tumor is still rather distant from the neurovascular bundles. Future research could therefore include the location of the tumor in relation to other nearby relevant structures such as the neurovascular bundles, bladder neck, urethra, and urinary sphincter. 

The AUC of the models ranged from 0.86 to 0.91 at the lesion-specific level, which is higher than the whole-gland EPE prediction based on 3T mpMRI data previously evaluated, which showed AUC values between 0.728 and 0.883 [9,10,12]. Xu et al. [11] also performed a lesion-specific approach with mostly texture-based features and obtained an 81.8% accuracy on a testing cohort of 33 lesions. Here, we not only significantly increased the amount of data in the training and testing cohorts, but also included geometric-based features. Geometric-based features have the additional benefit of enabling 3D visualization which can potentially be useful for patient communication and surgical planning. Our study demonstrates the RF model’s robustness in handling small geometric changes and avoiding overfitting, but it remains crucial to validate the predicted values when slightly altering the geometric variables to ensure the reliability of the model’s predictions. It should be further established how the prediction model can be implemented in clinical practice, possibly by visualizing segmentations and associated predicted values for each lesion.

To our knowledge, this study is the first to present an externally validated radiomics-based predictive model with novel radiomics (i.e., TCSA and TCV) at a lesion-specific level that demonstrates model reproducibility. RF feature importance scores showed that the TCL, lesion diameter, lesion volume, and TCSA were the most important features. These novel geometric radiomics have proven to outperform these known radiomics, possibly due to the three-dimensional, and thus more accurate, quantification. Currently, radiological assessment of EPE is based on several imaging features, including a tumor-capsule interface greater than 10 mm or 15 mm, a bulging prostatic contour, and a macroscopic breach of the capsule [24]. The TCSA is not included in this assessment and our results indicated that adding this novel radiomic feature next to conventional features improved EPE prediction.

Despite the relevance of the findings, some important limitations need to be acknowledged. Firstly, this study comprised retrospective data, indicating there was no central re-evaluation of MRI or pathology data. We included ISUP biopsy grades from systematic and targeted biopsies, but ISUP biopsies should correspond to separable suspicious lesions visible on MRI in prediction models for a more accurate representation at a lesion-specific level. Secondly, a single observer performed the semi-automated segmentations with supervision, while radiomics might be sensitive to the specific segmentation method used. However, no significant differences would be expected with multiple observers because the use of AI for assisted prostate and lesion segmentations tends to decrease the interrater variability, and the same suspicious lesions as in the radiology reporting were included. Thirdly, the extraction of radiomics in this study was performed using commercial software that is certified for clinical use but may not be widely available in all centers. Finally, prospective data is needed to evaluate whether AI-powered radiomic models can lead to more accurate treatment assignments and improved clinical outcomes. 

## 5. Conclusions

This study demonstrates that the use of AI algorithms provides high diagnostic performance in predicting EPE based on MRI data. Specifically, the assessment of tumor contact surface area in addition to the contact length and lesion diameter shows an improvement in the prediction of EPE. Our externally validated prediction model yields a greater likelihood of correctly detecting EPE lesions compared with radiological reporting and is the first step toward lesion-specific treatment assignment.

## Figures and Tables

**Table 1:** Patient characteristics of the overall study cohort, training cohort, internal test cohort, and external test cohort.

	Overall Cohort (n = 794)	Training Cohort (n = 524)	Internal Test Cohort (n = 131)	External Test Cohort (n = 139)
Age (yr), median (IQR)	67 (63–71)	67 (62–71)	67 (64–71)	67 (63–70)
PSA (ng/mL), median (IQR)	8.0 (5.8–12.1)	8.3 (6.0–12.6)	7.6 (5.4–12)	7.8 (5.6–10.8)
Biopsy ISUP grade group, n (%)				
1	167 (21.0)	111 (21.2)	33 (25.2)	23 (16.5)
2	285 (35.9)	175 (33.4)	49 (37.4)	61 (43.9)
3	148 (18.6)	99 (18.9)	21 (16.0)	28 (20.1)
4	125 (15.7)	91 (17.4)	17 (13.0)	17 (12.2)
5	69 (8.7)	48 (9.2)	11 (8.4)	10 (7.2)
Biopsy type				
Systematic	279 (35.1)	214 (40.8)	47 (35.9)	18 (12.9)
Target	515 (64.9)	310 (59.2)	84 (64.1)	121 (87.1)
Radiological T stage, n (%)				
T2	487 (61.3)	342 (65.3)	78 (59.5)	67 (48.2)
T3	306 (38.5)	181 (34.5)	53 (40.5)	72 (51.8)
T4	1 (0.1)	1 (0.2)	0 (0)	0 (0)
Pathological T stage, n (%)				
T2	420 (52.9)	281 (53.6)	70 (53.4)	69 (49.6)
T3	371 (46.7)	241 (46.0)	60 (45.8)	70 (50.4)
T4	3 (0.4)	2 (0.4)	1 (0.8)	0 (0)

Abbreviations: PSA = prostate specific antigen; IQR = interquartile range; ISUP = International Society of Urological Pathology.

**Table 2:** Performance results of internal and external test cohorts at lesion-specific level.

Model	Metric	Internal Test Cohort	External Test Cohort
Extra Trees	AUC	0.86 (0.80–0.92)	0.88 (0.83–0.93)
	Accuracy	0.81 (0.75–0.87)	0.81 (0.75–0.86)
	Sensitivity	0.78 (0.66–0.87)	0.79 (0.68–0.87)
	Specificity	0.83 (0.75–0.89)	0.82 (0.74–0.88)
	PPV	0.73 (0.62–0.83)	0.73 (0.62–0.81)
	NPV	0.87 (0.79–0.92)	0.87 (0.79–0.92)
Random Forest	AUC	0.86 (0.80–0.92)	0.88 (0.83–0.93)
	Accuracy	0.80 (0.73–0.85)	0.83 (0.76–0.87)
	Sensitivity	0.72 (0.59–0.81)	0.72 (0.60–0.81)
	Specificity	0.84 (0.76–0.90)	0.89 (0.82–0.93)
	PPV	0.73 (0.60–0.83)	0.80 (0.68–0.88)
	NPV	0.83 (0.75–0.89)	0.84 (0.77–0.89)
Logistic Regression	AUC	0.87 (0.81–0.93)	0.91 (0.87–0.95)
	Accuracy	0.80 (0.73–0.86)	0.83 (0.76–0.87)
	Sensitivity	0.77 (0.65–0.86)	0.65 (0.53–0.75)
	Specificity	0.82 (0.74–0.89)	0.93 (0.87–0.97)
	PPV	0.72 (0.60–0.81)	0.85 (0.73–0.92)
	NPV	0.86 (0.77–0.91)	0.81 (0.74–0.87)

Abbreviations: AUC = area under the curve; PPV = positive predictive value; NPV = negative predictive value. Data was presented as mean and 95% confidence interval.

**Table 3:** Radiology performance at lesion-specific level.

Metric	Internal Test Cohort	External Test Cohort
Accuracy	0.71 (0.64–0.77)	0.67 (0.60–0.73)
Sensitivity	0.53 (0.41–0.65)	0.65 (0.53–0.75)
Specificity	0.81 (0.73–0.88)	0.69 (0.60–0.76)
PPV	0.63 (0.49–0.75)	0.55 (0.45–0.66)
NPV	0.75 (0.66–0.82)	0.76 (0.67–0.83)

Abbreviations: PPV = positive predictive value; NPV = negative predictive value.

## Data Availability

The data presented in this study are available on request from the corresponding author. The data are not publicly available due to privacy and ethical reasons.

## Supplementary Materials

The following supporting information can be downloaded at: https://www.mdpi.com/article/10.3390/cancers15225452/s1, Figure S1: Included features of the random forest model after impurity-based feature reduction; Table S1: MRI sequence parameters of T2-weighted imaging and diffusion-weighted imaging of the training cohort; Table S2: MRI-based radiomics in this study; Table S3: Performance results of internal and external test cohorts with original and varied geometric variables (5% larger and 5% smaller) at lesion-specific level.
