# Supplementary material for: The Development and External Validation of Artificial Intelligence-Driven MRI-Based Models to Improve Prediction of Lesion-Specific Extraprostatic Extension in Patients with Prostate Cancer
Source: Cancers (Basel). 2023 Nov 17;15(22):5452. doi: 10.3390/cancers15225452 (PMC10670855; doi:10.3390/cancers15225452)
Supplement: Supplementary file 1 [file cancers-15-05452-s001.zip › cancers-2702793-supplementary.pdf]

Table S1. MRI sequence parameters of T2-weighted imaging and diffusion-weighted imaging of the training cohort.

	St. Antonius Hospital, Utrecht (n = 357)		St. Antonius Hospital, Nieuwegein (n = 16)		Canisius Wilhelmina Hospital (n = 151)	
MRI System	Siemens 3T		Philips 3T		Philips 3T	
Sequence	T2w	DWI	T2w	DWI	T2w	DWI
TR (ms)	5000	3500	4207	3000<br>b-1400: 3500	3600	3618
TE/TEeq (ms)	103	63	110/78	84<br>b-1400: 87	120/100	73
Excitation/refocusing flip angle (°)	90/150	90/180	90/110	90/180	90/variable	90/180
Echo train length/EPI factor	25	72	29	61	18	61
FOV (mm)	200	256	200	160	200	200
Slice orientation	Axial, coronal, sagittal	Axial	Axial, coronal, sagittal	Axial	Axial, coronal, sagittal	Axial
Acquired voxel size (mm)	0.78 × 0.63	2.0 × 2.0	0.78 × 0.63	2.0 x 2.0	0.4 × 0.55	2.0 × 2.0
Reconstructed voxel size (mm)	0.63 × 0.63	2.0 × 2.0	0.63 × 0.63	2.0 x 2.0<br>b-1400: 2.5 x 2.5	0.3 × 0.3	1.8 × 1.8
Slice thickness (mm)	3.0	3.0	3.0	3.0	3.0	4.0
Number of slices (axial)	26	26	26	26	26	20
b-values (s/mm^2)	-	50-800 (acquired)<br>- 1400 (calculated)	-	0-50-400-800-1400	-	0-800-1400
Number of averages (per b-value)	2	5-10	1	2-2-3-5-3	2	3-10-10
Acquisition time (min:sec)	3:30	2:57	2:28	3:18<br>b-1400: 3:16	5:04	3:55
Parallel imaging acceleration factor	2	2	1.8	3	1	2.5

Table S2. MRI-based radiomic features in this study

Radiomics	Unit	Definition
Average ADC lesion inside	$\mu m^2/s$	Average apparent diffusion coefficient of lesion located in the prostate.
Average ADC lesion outside	$\mu m^2/s$	Average apparent diffusion coefficient of lesion part beyond the prostate capsule.
Average ADC prostate	$\mu m^2/s$	Average apparent diffusion coefficient of the prostate.
Average high b value lesion inside	s/mm^2	Average of high b-value intensities of lesion located in the prostate.
Average high b value lesion outside	s/mm^2	Average of high b-value intensities of lesion part beyond the prostate capsule.
Average high b value prostate	s/mm^2	Average of high b-value intensities of the prostate.
Axial lesion diameter	mm	Maximum lesion diameter across all slices in the axial plane.
Axial TCL	mm	Maximum contact length between the prostate capsule and lesion across all slices in the axial plane.
Axial TCSA	mm^2	Sum of all per-slice axial tumour contact lengths.
Coronal lesion diameter	mm	Maximum lesion diameter across all slices in the coronal plane.
Coronal TCL	mm	Maximum contact length between the prostate capsule and lesion across all slices in the coronal plane.
Coronal TCSA	mm^2	Sum of all per-slice coronal tumour contact lengths.
Lesion volume	mm^3	Lesion volume (lesion volume inside + lesion volume outside).
Lesion volume inside	mm^3	Volume of lesion located in the prostate.
Lesion volume outside / Radial volume	mm^3	Volume of lesion part beyond the prostate capsule.
Lesion zone	Category	▪ Peripheral zone apex<br>▪ Peripheral zone mid<br>▪ Peripheral zone base<br>▪ Transition zone
Maximum ADC lesion inside	$\mu m^2/s$	Maximum apparent diffusion coefficient of lesion located in the prostate.
Maximum ADC lesion outside	$\mu m^2/s$	Maximum apparent diffusion coefficient of lesion part beyond the prostate capsule.
Maximum ADC prostate	$\mu m^2/s$	Maximum apparent diffusion coefficient of the prostate.
Maximum high b value lesion inside	s/mm^2	Maximum of high b-value intensities of lesion located in the prostate.
Maximum high b value lesion outside	s/mm^2	Maximum of high b-value intensities of lesion part beyond the prostate capsule.
Maximum high b value prostate	s/mm^2	Maximum of high b-value intensities of the prostate.
Median ADC lesion inside	$\mu m^2/s$	Median apparent diffusion coefficient of lesion located in the prostate.
Median ADC lesion outside	$\mu m^2/s$	Median apparent diffusion coefficient of lesion part beyond the prostate capsule.
Median ADC prostate	$\mu m^2/s$	Median apparent diffusion coefficient of the prostate.
Median high b value lesion inside	s/mm^2	The median of high b-value intensities of lesion located in the prostate.
Median high b value lesion outside	s/mm^2	Median of high b-value intensities of lesion part beyond the prostate capsule.
Median high b value prostate	s/mm^2	Median of high b-value intensities of the prostate.
Minimum ADC lesion inside	$\mu m^2/s$	Minimum apparent diffusion coefficient of lesion located in the prostate.
Minimum ADC lesion outside	$\mu m^2/s$	Minimum apparent diffusion coefficient of lesion part beyond the prostate capsule.
Minimum ADC prostate	$\mu m^2/s$	Minimum apparent diffusion coefficient of the prostate.

Minimum high b value lesion inside	s/mm^2	Minimum of high b-value intensities of lesion located in the prostate.
Minimum high b value lesion outside	s/mm^2	Minimum value of high b-value intensities of lesion part beyond the prostate capsule.
Minimum high b value prostate	s/mm^2	Minimum of high b-value intensities of the prostate.
Minimum 2D distance	mm	Minimum distance of the lesion boundary to the prostate capsule across all slices in the axial plane.
Minimum 3D distance	mm	Minimum distance of the lesion boundary to the prostate capsule across all planes.
Prostate volume	mm^3	Prostate volume.
PSA density	ng/ml^2	PSA value / prostate volume.
Radial distance	mm	Maximum perpendicular distance between the prostate capsule and lesion part beyond the prostate across all slices in the axial plane.
Sagittal lesion diameter	mm	Maximum lesion diameter across all slices in the sagittal plane.
Sagittal TCL	mm	Maximum contact length between the prostate capsule and lesion across all slices in the sagittal plane.
Sagittal TCSA	mm^2	Sum of all per-slice sagittal tumour contact lengths.
SD ADC lesion inside	μm^2/s	Standard deviation apparent diffusion coefficient of lesion in the prostate.
SD ADC lesion outside	μm^2/s	Standard deviation apparent diffusion coefficient of lesion part beyond the prostate capsule.
SD ADC prostate	μm^2/s	Standard deviation apparent diffusion coefficient of the prostate.
SD high b value lesion inside	s/mm^2	Standard deviation of high b-value intensities of lesion located in the prostate.
SD high b value lesion outside	s/mm^2	Standard deviation of high b-value intensities of lesion part beyond the prostate capsule.
SD high b value prostate	s/mm^2	Standard deviation of high b-value intensities of the prostate.
TCSA (1 – 5 mm)	mm^2	Contact surface area using the Euclidean distances between the lesion surface and prostate capsule within 1 to 5 mm.
Tumor contact volume (TCV)	mm^3	Interaction volume between the prostate capsule and lesion.
TCV (1 – 5 mm)	mm^3	Contact volume using the Euclidean distances between the lesion and prostate capsule within 1 to 5 mm.
Q1 ADC lesion inside	μm^2/s	First quartile (25th percentile) apparent diffusion coefficient of lesion located in the prostate.
Q1 ADC lesion outside	μm^2/s	First quartile (25th percentile) apparent diffusion coefficient of lesion part beyond the prostate capsule.
Q1 ADC prostate	μm^2/s	First quartile (25th percentile) apparent diffusion coefficient of the prostate.
Q1 high b value lesion inside	s/mm^2	First quartile (25th percentile) of high b-value intensities of lesion located in the prostate.
Q1 high b value lesion outside	s/mm^2	First quartile (25th percentile) of high b-value intensities of lesion part beyond the prostate capsule.
Q1 high b value prostate	s/mm^2	First quartile (25th percentile) of high b-value intensities of the prostate.
Q3 ADC lesion inside	μm^2/s	Third quartile (75th percentile) apparent diffusion coefficient of lesion located in the prostate.
Q3 ADC lesion outside	μm^2/s	Third quartile (75th percentile) apparent diffusion coefficient of lesion part beyond the prostate capsule.
Q3 ADC prostate	μm^2/s	Third quartile (75th percentile) apparent diffusion coefficient of the prostate.

Q3 high b value lesion inside	s/mm^2	Third quartile (75th percentile) of high b-value intensities of lesion located in the prostate.
Q3 high b value lesion outside	s/mm^2	Third quartile (75th percentile) of high b-value intensities of lesion part beyond the prostate capsule.
Q3 high b value prostate	s/mm^2	Third quartile (75th percentile) of high b-value intensities of the prostate.

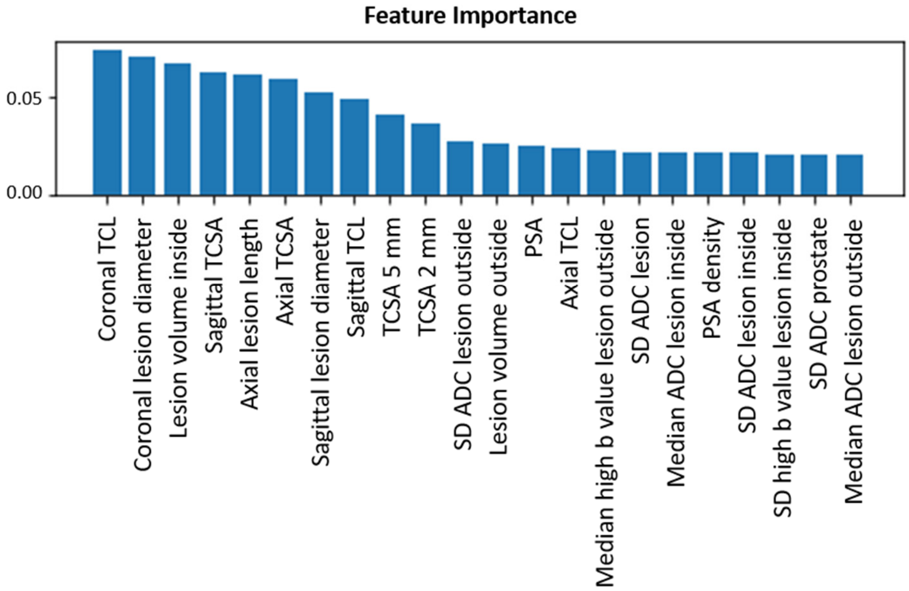

Figure S1. Included features of the random forest model after impurity-based feature reduction.

Table S3. Performance results of internal and external test cohorts with original and varied geometric variables (5% larger and 5% smaller) at lesion-specific level.

Model	Metric	Internal test cohort			External test cohort		
		Original	Larger	Smaller	Original	Larger	Smaller
Extra Trees	AUC	0.86 (0.80 – 0.92)	0.85 (0.79 – 0.91)	0.86 (0.80 – 0.92)	0.88 (0.83 – 0.93)	0.88 (0.83 – 0.93)	0.88 (0.83 – 0.93)
	Accuracy	0.81 (0.75 – 0.87)	0.80 (0.73 – 0.86)	0.79 (0.72 – 0.85)	0.81 (0.75 – 0.86)	0.79 (0.73 – 0.85)	0.80 (0.74 – 0.85)
	Sensitivity	0.78 (0.66 – 0.87)	0.78 (0.66 – 0.87)	0.70 (0.57 – 0.80)	0.79 (0.68 - 0.87)	0.82 (0.71 – 0.89)	0.76 (0.65 – 0.84)
	Specificity	0.83 (0.75 – 0.89)	0.81 (0.73 – 0.88)	0.84 (0.76 – 0.90)	0.82 (0.74 – 0.88)	0.78 (0.70 – 0.84)	0.83 (0.75 – 0.89)
	PPV	0.73 (0.62 – 0.83)	0.71 (0.59 – 0.81)	0.72 (0.60 – 0.82)	0.73 (0.62 – 0.81)	0.69 (0.59 – 0.78)	0.73 (0.62 – 0.82)
	NPV	0.87 (0.79 – 0.92)	0.86 (0.78 – 0.92)	0.83 (0.74 – 0.89)	0.87 (0.79 – 0.92)	0.88 (0.80 – 0.93)	0.85 (0.78 – 0.91)
Random Forest	AUC	0.86 (0.80 – 0.92)	0.86 (0.80 – 0.92)	0.85 (0.79 – 0.91)	0.88 (0.83 – 0.93)	0.88 (0.83 – 0.93)	0.88 (0.83 – 0.93)
	Accuracy	0.80 (0.73 – 0.85)	0.80 (0.73 – 0.85)	0.80 (0.73 – 0.85)	0.83 (0.76 – 0.87)	0.81 (0.75 – 0.86)	0.81 (0.75 – 0.86)
	Sensitivity	0.72 (0.59 – 0.81)	0.75 (0.63 – 0.84)	0.72 (0.59 – 0.81)	0.72 (0.60 – 0.81)	0.73 (0.62 – 0.82)	0.69 (0.58 – 0.79)
	Specificity	0.84 (0.76 – 0.90)	0.82 (0.74 – 0.89)	0.84 (0.76 – 0.90)	0.89 (0.82 – 0.93)	0.86 (0.78 – 0.91)	0.88 (0.81 – 0.93)
	PPV	0.73 (0.60 – 0.83)	0.71 (0.59 – 0.81)	0.73 (0.60 – 0.83)	0.80 (0.68 – 0.88)	0.75 (0.64 – 0.84)	0.78 (0.66 – 0.86)
	NPV	0.83 (0.75 – 0.89)	0.85 (0.76 – 0.91)	0.83 (0.75 – 0.89)	0.84 (0.77 – 0.89)	0.84 (0.77 – 0.90)	0.83 (0.75 – 0.88)
Logistic Regression	AUC	0.87 (0.81 – 0.93)	0.87 (0.81 – 0.93)	0.87 (0.81 – 0.93)	0.91 (0.87 – 0.95)	0.91 (0.87 – 0.95)	0.91 (0.87 – 0.95)
	Accuracy	0.80 (0.73 – 0.86)	0.80 (0.73 – 0.85)	0.80 (0.73 – 0.85)	0.83 (0.76 – 0.87)	0.83 (0.76 – 0.87)	0.81 (0.75 – 0.86)
	Sensitivity	0.77 (0.65 0.86)	0.80 (0.68 – 0.88)	0.72 (0.59 – 0.81)	0.65 (0.53 – 0.75)	0.70 (0.59 – 0.80)	0.58 (0.46 – 0.69)
	Specificity	0.82 (0.74 – 0.89)	0.79 (0.71 – 0.86)	0.84 (0.76 – 0.90)	0.93 (0.87 – 0.97)	0.90 (0.83 – 0.87)	0.95 (0.89 – 0.98)
	PPV	0.72 (0.60 – 0.81)	0.70 (0.58 – 0.79)	0.73 (0.60 – 0.83)	0.85 (0.73 – 0.92)	0.81 (0.69 – 0.89)	0.87 (0.75 – 0.94)
	NPV	0.86 (0.77 – 0.91)	0.87 (0.79 – 0.92)	0.83 (0.75 – 0.89)	0.81 (0.74 – 0.87)	0.83 (0.76 – 0.89)	0.79 (0.71 – 0.85)

Abbreviations: AUC = area under the curve; PPV = positive predictive value; NPV = negative predictive value. Data was presented as mean and 95% confidence interval.
